# Supplementary material for: Influencing Activity of Bats by Dimly Lighting Wind Turbine Surfaces with Ultraviolet Light
Source: Animals (Basel). 2021 Dec 21;12(1):9. doi: 10.3390/ani12010009 (PMC8744972; doi:10.3390/ani12010009)
Supplement: Supplementary file 1 [file animals-12-00009-s001.zip › animals-1461752-supplementary/revised_supplementary_files/SUPPEMENTARY_INFO_CAPTIONS_Cryan et al.docx]

**Supplementary Information Captions.**

**Any use of trade, firm, or product names is for descriptive purposes only and does not imply endorsement by the U.S. Government.**

**Figure S1.** ***Spectrophotometer Readings of Ultraviolet (UV) Lights.*** Illumination intensity of three UV illuminators at various distances measured by spectrophotometer during off-turbine ground testing. We used a portable USB spectrophotometer sensitive to light ranging from 225 to 1,000 nm (Model AFBR-S20M2WU, Broadcom Inc., San Jose, California, USA) to measure light from the array of illuminators mounted 2 m above the ground and pointed down a dark residential street at night with no moon. We fit the input tube of the spectrophotometer with a dichroic filter identical to the type used for our illuminators, which prevented light at wavelengths >400 nm from reaching the sensor. We pointed the filtered spectrophotometer sensor away from the light source and measured illumination intensity reflecting off a neutral gray camera calibration card mounted 10 cm away and facing the illuminators. We took spectrophotometer readings at distances ranging from 75 to 5 m, at 10 m intervals as measured with a laser rangefinder (Model ProStaff 1000, Nikon Corporation, Minato, Japan). Manufacturer’s software (Waves) recorded measurements as normalized counts averaged over a 1-s sampling period, in units of µ/cm2/nm; we baseline-corrected those values with the “normal” function of the Waves software and then approximated illumination intensity of the cast light by integrating the area under the curve for readings between 300 and 500 nm.

**Figure S2.** ***Actual and Linearly Interpolated Value (ALIV) Plots.*** Response to UV illumination on nights with (purple circles) and without (open circles) UV treatment based on counts of bats (all detections, cumulative duration of detections, and high-risk detections), insects, birds, and “high-flyer”. We show ALIV comparisons for both the spring (upper sub-panel) and autumn (lower sub-panel) periods of monitoring. The difference between nightly observed versus interpolated values are indicated with green arrows if the treatment effect reduced expected counts and with red arrows if it increased expected counts. ‘Duration’ refers to the cumulative duration of detected bat flight in seconds.

**Video S1.** ***North Turbine Imaged from Approximately 1.5 km to the Northeast.*** We used a scientific-grade, high-sensitivity, high-resolution digital imaging camera (Teledyne Photometrics, Tucson, Arizona, USA). Camera exposure time was set to 250 ms and lens was fitted with a pass filter that only allowed UV parts of the light spectrum to pass, with a 50% transmission peak at 365 nm, tapering off the peak to <25% by 340 nm and 380 nm.

**Video S2.** ***North Turbine Imaged from Approximately 1.5 km to the Southwest.*** Methods as described in legend of Video S1.

**Video S3. *North Turbine Imaged from Approximately 5.0 km to the Southwest.*** Methods as described in legend of Video S1.

**Video S4***.* ***Time Lapse of Long-exposure Camera Imagery Taken Most Nights of the Study Within an Hour of Midnight.*** Illumination of turbines with ultraviolet light can be seen alternating between the North Turbine (Left) and South Turbine (right). The first three-quarters of the video show landscapes using a camera lens that passed both near-UV and near-infrared light, then for the remainder of video shows the scene with a filter that only passed UV. Nights when the camera malfunctioned and did not record imagery show a black blank screen for 1-s in this video.
