# Supplementary figures and images for: Influencing Activity of Bats by Dimly Lighting Wind Turbine Surfaces with Ultraviolet Light
Source: Animals (Basel). 2021 Dec 21;12(1):9. doi: 10.3390/ani12010009 (PMC8744972; doi:10.3390/ani12010009)

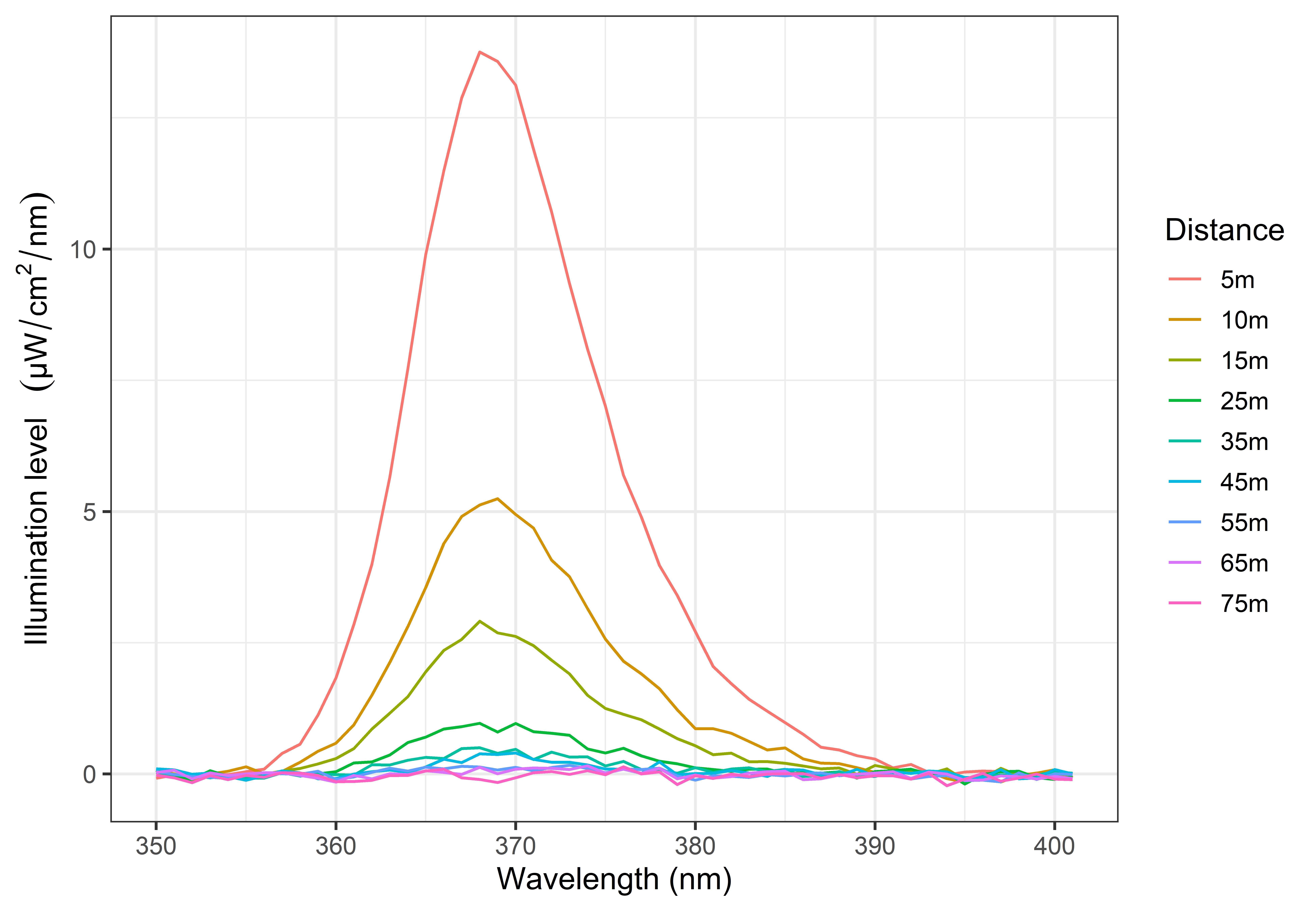

Supplement: Supplementary file 1 [file animals-12-00009-s001.zip › animals-1461752-supplementary/revised_supplementary_files/Figure S1.jpg]

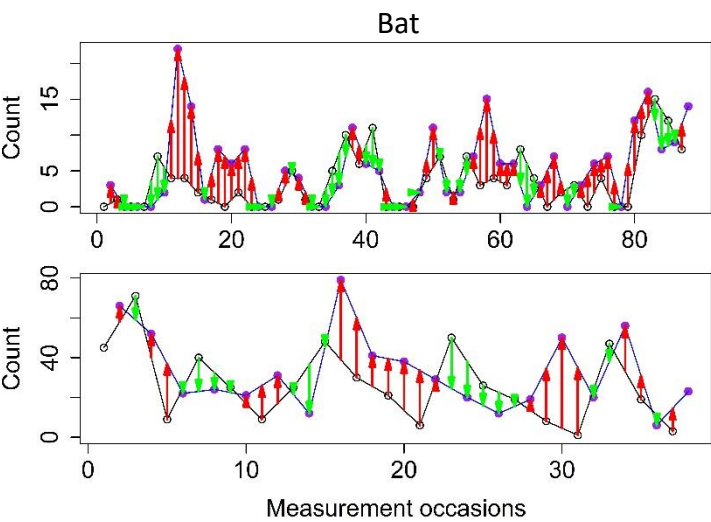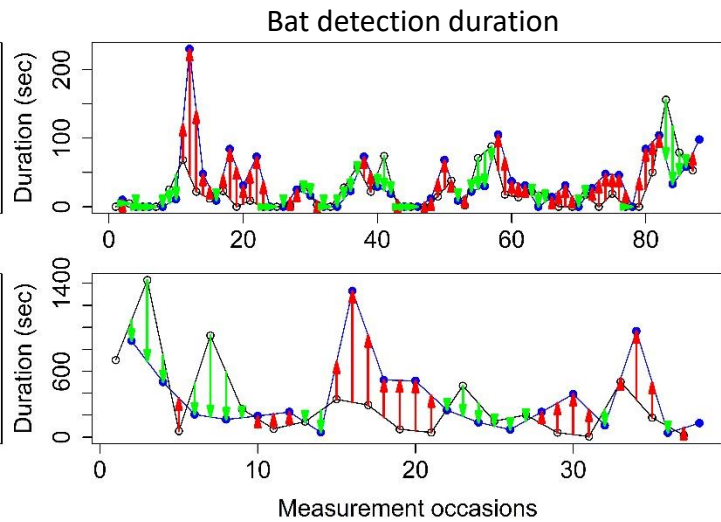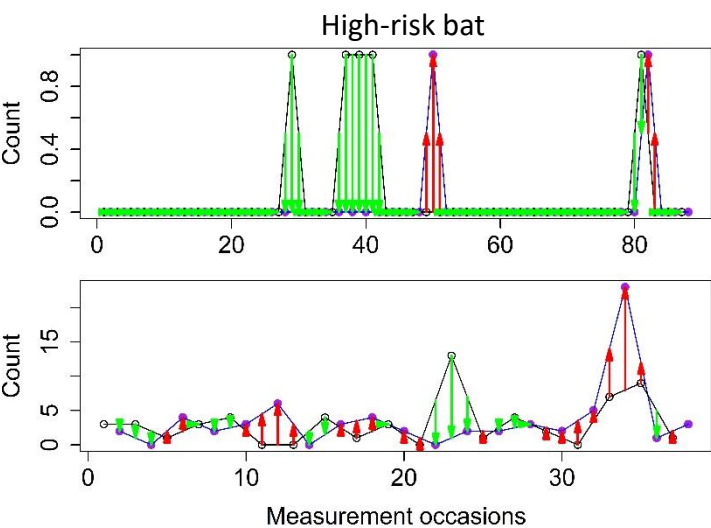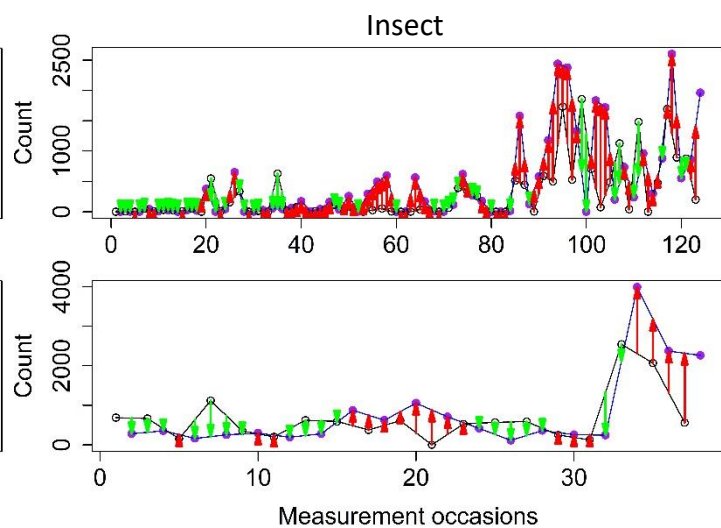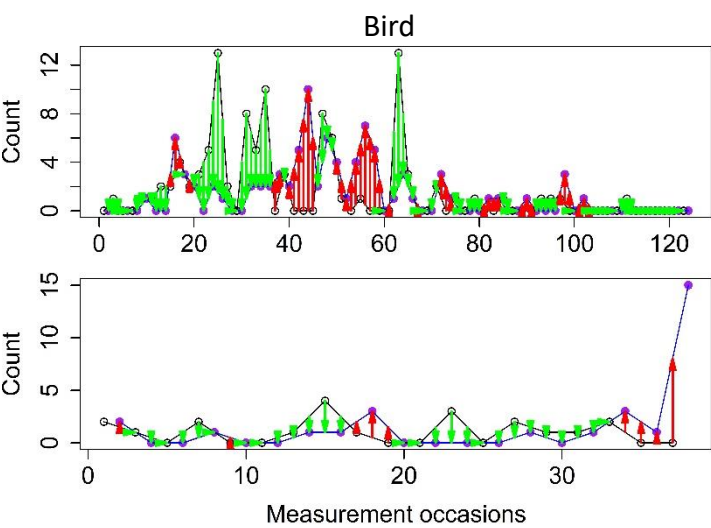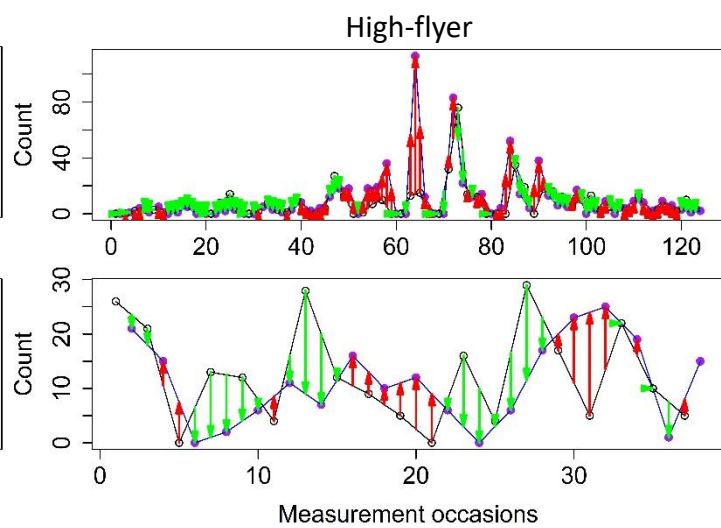

Supplement: Supplementary file 1 [file animals-12-00009-s001.zip › animals-1461752-supplementary/revised_supplementary_files/Figure S2.pdf]
